# Scalable transcriptomics analysis with Dask: applications in data science and machine learning

**DOI:** 10.1186/s12859-022-05065-3

**Published:** 2022-11-30

**Authors:** Marta Moreno, Ricardo Vilaça, Pedro G. Ferreira

**Affiliations:** 1 Department of Computer Science, Faculty of Sciences, University of Porto, Rua do Campo Alegre, 4169-007 Porto, Portugal; 2 Laboratory of Artificial Intelligence and Decision Support, INESC TEC, Rua Dr. Roberto Frias, 4200-465 Porto, Portugal; 3 Institute of Molecular Pathology and Immunology of the University of Porto, Institute for Research and Innovation in Health (i3s), R. Alfredo Allen 208, 4200-135 Porto, Portugal; 4 High-Assurance Software Laboratory, INESC TEC, Rua Dr. Roberto Frias, 4200-465 Porto, Portugal; 5 Department of Informatics, Minho Advanced Computing Center, University of Minho, Gualtar, 4710-070 Braga, Portugal

**Keywords:** Machine learning, Scalable data science, Gene expression, Transcriptomics, Data analysis

## Abstract

**Background:**

Gene expression studies are an important tool in biological and biomedical research. The signal carried in expression profiles helps derive signatures for the prediction, diagnosis and prognosis of different diseases. Data science and specifically machine learning have many applications in gene expression analysis. However, as the dimensionality of genomics datasets grows, scalable solutions become necessary.

**Methods:**

In this paper we review the main steps and bottlenecks in machine learning pipelines, as well as the main concepts behind scalable data science including those of concurrent and parallel programming. We discuss the benefits of the *Dask* framework and how it can be integrated with the Python scientific environment to perform data analysis in computational biology and bioinformatics.

**Results:**

This review illustrates the role of *Dask* for boosting data science applications in different case studies. Detailed documentation and code on these procedures is made available at https://github.com/martaccmoreno/gexp-ml-dask.

**Conclusion:**

By showing when and how *Dask* can be used in transcriptomics analysis, this review will serve as an entry point to help genomic data scientists develop more scalable data analysis procedures.

## Background

Tissue homeostasis results from an intricate gene regulation network that when disrupted can lead to disease. Gene expression is an intermediate state linking the genome to phenotypic outcomes. Biomedical studies have relied on the analysis of gene expression levels to disease state, progression, outcome and treatment [1–3]. Changes in gene expression have helped identify patterns and signatures associated with disease (e.g. community-acquired pneumonia [4] or tuberculosis [5]), improve the understanding of aging conditions [6, 7] and complement genetic information in Mendelian disease diagnosis [8]. Furthermore, analyzing these alterations can facilitate the discovery, development and assessment of novel drug treatments [9–11], the optimization of drug application and dosage in cancer therapies [12, 13], or the prediction of response to therapy [3].

Advances in sequencing technologies have produced an unprecedented amount of multi-modal molecular data. This opened the possibility for a thorough and informative interrogation of genotype-phenotype associations on complex diseases, presenting new opportunities for precision medicine. However, finding statistically significant and meaningful associations between molecular data and phenotypic or clinical annotations remains a hard challenge. This difficulty is exacerbated by the high dimensionality of the data and, as is the case with gene expression, a considerable stochastic component.

**Fig. 1:**
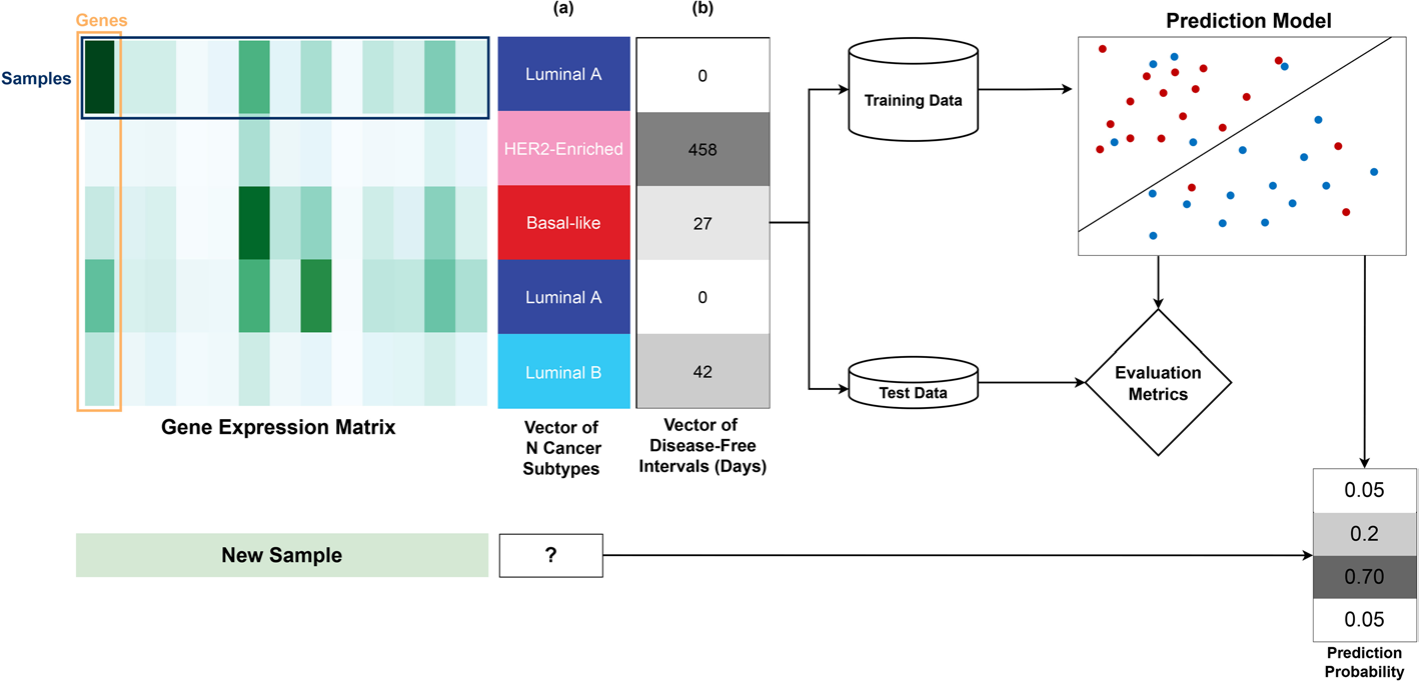
Supervised learning for predicting cancer-related phenotypes from gene expression data. **a** Classification identifies target labels, including cancer subtypes; **b** regression can predict progression and outcome measures, such as disease-free intervals. After the data is partitioned into training and test sets, a ML algorithm is fit on the training data. The model is evaluated using hold-out test data

Machine Learning (ML) seeks to automatically capture statistical associations in data while improving knowledge with additional evidence [14–16]. ML is of particular interest in computational biology because it can describe biological phenomena without the need to explicitly model them. Supervised ML builds mathematical models that map the input data, described by several attributes or features, to the corresponding output value or label for each instance or sample. The model can then be used to predict the outcome of unseen or incoming data. Figure 1 shows a general flowchart of a supervised learning (SL) pipeline: (1) train/test data split; (2) model training; (3) model evaluation. Discrete categories are determined through classification tasks (Fig. 1a); when predicting numerical values, the task is called regression (Fig. 1b).

In the context of gene expression analysis, there are several examples of phenotype inference from predictive models. Classification has been used for predicting therapeutic or drug response [17, 18], as well as cancer molecular subtype from gene expression, DNA methylation data, or both [19]. Use cases for regression include the prediction of expression levels of target genes from landmark genes [20] or mutational effects from DNA sequences [21].

While ML provides an adequate solution for statistical association challenges, it often incurs substantial computational demands. Processing power and memory requirements are amplified by the size of the data and the complexity of the models. When building models in a single machine, such as a laptop, it might be too costly or even impossible to load large amounts of data into memory. Moreover, while high-performance computing (HPC) solutions are capable of meeting these requirements, it is not always trivial to fully leverage these resources. Therefore, implementations for scalable computing of ML and other data analytics tasks are required.

There are several frameworks for scalable data analytics available for the widely-used Python programming language. In this review we will focus on a particular framework called *Dask* [22]. *Dask* divides data into smaller blocks on which to perform highly parallel computations, thus allowing larger data sets to fit in the memory of single machines. It tightly integrates with existing libraries for Python data analytics and shares a similar interface for implementation, which minimizes the need for code rewrites and facilitates the transition to HPC environments. Here we explore the potential of *Dask* for scalable ML and data science, applied to bulk and single cell transcriptomics, providing usage recommendations supported by numerous code examples and performance tests.

This paper begins with a review on the need for scalable data analysis in computational biology. We start by describing the standard supervised ML workflow and how it can be adapted for gene expression data analysis. In the following section, we look into the specifics of the Python programming language and how it can be used for ML and data science, discussing its advantages and limitations. We then introduce the general concepts of distributed and parallel computing and enumerate different scalable ML frameworks, with a focus on *Dask*. We perform several benchmarks and comparisons highlighting the advantages of *Dask*. Finally, we propose guidelines for the efficient use of *Dask* for transcriptomics analysis. This review is supported by examples of different tasks, comparative performance tests, and supporting code.

### Building gene expression predictive models

Gene expression predictive models are built using supervised learning, which often rely on the data being stored in a structured format, such as tables, see Fig. 1. The labels (categorical or numerical) represent the phenotype or the clinical information to be inferred.

Supervised learning pipelines encompass several key steps [23]. First, gene expression and phenotype data are loaded. At this point, preprocessing can be applied to prepare the data for the remaining steps. The data is then split into training and test sets. ML algorithms learn from the training data to generate a predictive model. In supervised learning, training consists in finding the coefficients, known as model parameters, that provide the best mapping between input features and output labels, as judged by intermediate validation scores. Lastly, the trained model is evaluated on the hold-out test set. The test set is used to estimate the generalization error, *i.e.* how well the model behaves on unseen data. To ensure the robustness of the generalization error, k-fold cross-validation (CV) [24, 25] is customarily performed during model training. This strategy consists in creating several (*k*) train/test partitions from the original dataset, repeating the model-building process *k* times, and averaging out *k* evaluation scores. If the model reaches an evaluation score that is deemed sufficiently high for a specific application, it can be deployed to work with new incoming data.

For the most part, the development of transcriptomics predictive models follows the traditional approach. However, because gene expression levels obtained from RNA-seq experiments are noisy and have a large amplitude, data preprocessing may have an impact on downstream results. Therefore, feature selection, scaling and normalization are required [26–32]. Feature selection removes less informative genes, for example those with low variability or low expression levels. It can also select biologically-relevant gene subsets (e.g. protein coding). Scaling is used to attenuate fold differences in expression ranges, typically by shifting the data from a linear to a logarithmic scale. Sample normalization methods account for differences that arise from the sequencing process, like library size, batch effects and gene structure.

Although models are trained automatically, the building process can be controlled by static values defined *a priori* by the user, known as hyper-parameters [33]. Different hyper-parameter values influence the performance of the final predictive model in different ways. Therefore, hyper-parameter optimization (HPO) can be performed during CV in an attempt to lower the generalization error. If the model reaches an optimized performance according to the evaluation measures, the model is selected and applied to new incoming data. Determining the best set of hyper-parameter values is a computationally expensive combinatorial problem. It requires traversing a multidimensional grid of values, with a model to be trained and tested for each combination. Random search alleviates this issue by sampling a limited number of hyper-parameter combinations [34].

To accurately estimate the performance of an ML algorithm, k-fold CV and HPO can be combined into a strategy known as nested CV. While this provides exhaustive performance estimates, it further exacerbates the computational cost incurred by the two techniques in isolation.

### Scientific computing with Python

Python is an interpreted, high-level and general-purpose programming language with a focus on readability [35]. It offers interactive and scripting modes that allow for quick prototyping and deployment of a broad range of scientific applications. Data science and ML are areas where Python’s role has greatly expanded in recent years. The Scientific Python Environment (SPE) is at the core of this expansion. The SPE is based on several highly efficient libraries that support numerical and scientific computation, namely: *NumPy* [36], for storing and operating over large and multi-dimensional arrays and matrices; *Scipy* [37] for fundamental routines and algorithms in scientific and technical computation; *pandas* [38] for data manipulation and analysis; *matplotlib* [39] for plotting and visualization; and *scikit-learn* [40] for traditional ML. Python and the SPE have thus become popular tools for data science and ML [23]. However, as the size of the data increases and the tasks become more complex and expensive there is a need for improving program efficiency, namely in terms of execution runtime.

#### Python concurrent and parallel computation

Parallelism and concurrency are two approaches for improving the efficiency of a program. Their use is highly dependent on the underlying architecture of the central processing unit (CPU). In Python, they can be implemented through native packages such as *multiprocessing* and *multithreading*.

A *process* is an independent instance executed in a processor core or node with dedicated memory space. To speed up computation in CPU-intensive tasks, *multiprocessing* spawns multiple processes that execute parts of a task in parallel. A *thread* is run within a process and allows parts of a program to run concurrently when there is a separate flow of execution. Multiple threads can be run in the same process, sharing the same memory space. This reduces the overhead associated with copying and moving data, and making code faster and safer to execute. *Multithreading* allows concurrency by progressing multiple independent tasks simultaneously.

When multiple threads are launched at the same time, problems may arise if computations are performed out of order. Standard Python implementations address this issue by enforcing the global interpreter lock (GIL) mechanism. The GIL ensures that only a single thread is run at a time. To overcome this important limitation, it is necessary to find solutions for carrying out computations outside the GIL, either by rewriting code in a different language like C or using specialized libraries. In particular, SPE libraries implement several strategies for speeding up computation. For example, *NumPy* and *SciPy* can efficiently perform numerical linear algebra operations and bypass the GIL mechanism by leveraging a low-level Basic Linear Algebra Subprograms implementation known as OpenBLAS [41, 42]. SPE libraries also come with the native capability to spawn multiple processes, or jobs, to parallelize computation across several CPU cores. Yet, increasing the number of processes does not always speedup computation. In some cases, excessive parallelization can even be detrimental to overall performance. Therefore, understanding how multiprocessing processes might nest or interact with each other is critical for improving program performance.

#### Limitations of the scientific Python ecosystem

For all their promise, the advantages of parallelism can be difficult to achieve. To bypass the GIL, multiprocessing has to launch a Python instance containing a full, in-memory copy of the data for each additional CPU core in use. Furthermore, certain ML tasks such as nested CV or hyper-parameter optimization incur heavy computational burdens. Hence, the parallelization of ML pipelines rapidly fills available memory. Furthermore, increases in data size lead to difficulties in data processing and analysis. The limitations of a single machine become apparent when datasets no longer fit comfortably in memory or take too long to load and process. One approach to alleviate these issues would be to share the workload across several machines, but in Python this is not a straightforward task. Data science libraries like *pandas* or *scikit-learn* are not designed specifically to operate on distributed systems [43]. It is thus imperative to find computational paradigms that can handle multiple ML models simultaneously built from large or massive datasets and take full advantage of all available CPU cores through efficient parallelization, while offering the possibility to scale beyond a single machine.

### Scalable data science

The transformative power of data science is supported by advances in computing capabilities that enable the production, collection, storage and processing of increasingly larger amounts of data. Thus, the computational requirements of large datasets are an important bottleneck in data analysis. This bottleneck can be overcome by improving the quality and/or quantity of computational resources available to a single machine (scaling-up), or spreading computational load across several machines (scaling-out).

Scalable data analytics frameworks can assist in implementing these strategies, ensuring that available resources are fully and expanding computational capabilities in tasks such as ML. For example, when a dataset does not entirely fit in memory, out-of-core computation can be a solution. Out-of-core algorithms are optimized to efficiently fetch and transfer batches of data from disk to memory and to process them in smaller blocks, expanding total available memory.

For several years, the *Apache Spark* [44, 45] framework has been a popular choice for scalable data analytics. *Spark* is a unified engine written in Scala for distributed data processing. As a part of the all-in-one Apache ecosystem, *Spark* interfaces well with other Apache projects and is capable of integrating and parallelizing computation across several languages, namely Java, R, and Python (via the *Pyspark* library). However, integration with these languages requires additional programming efforts and often incurs serialization costs, as abstract data structures need to be converted between languages.

As such, several frameworks and tools are currently being developed under the Python ecosystem to scale one or several steps of data analysis across several CPUs (and, in some cases, GPUs) while minimizing the need for code rewrites (Table 1). Some frameworks focus on out-of-core parallelization of large tabular datasets, while others provide support for a complete analysis pipeline in a distributed environment. Overall, there is an ongoing effort to develop scalable data science frameworks that provide seamless integration and compatibility with existing SPE code. These frameworks aim to alleviate the burden associated with technical implementation, allowing researchers to focus on scientific questions.

From among the solutions for scalable data science in Python, this paper highlights the *Dask* framework [22].

### Scaling computational biology with *Dask*

*Dask* has been applied to a variety of scalable problems in computational biology. It has been used to introduce parallel computation in molecular dynamics analysis and simulations [46–49], efficiently handle genotype data as arrays [50], assess the reliability of methodologies for the analysis of human brain white matter connections [51], as a component in general neuroimagining pipelines [52], and for powering a workflow in gene regulatory network model exploration [53].

Applications of *Dask* specific to omics data analysis include scaling the reconstruction of gene regulatory networks from single-cell yeast RNA [54], integrative analysis of multi-omics data [55], inferring gene regulatory networks from large single-cell gene expression datasets [56, 57], analysis of high resolution rRNA sequencing data from multiple amplicons [58], and the study of tissue organization and cellular communications derived from spatial omics data analysis and visualization [59].

Furthermore, *Dask* plays a supporting role as an underlying component for several data science and machine learning tools used in computational biology. For example, the GPU-accelerated tool *RAPIDS* [60] builds on top of the *Dask* framework in order to scale its data preparation and model training steps across multiple GPUs and machines [23]. In this assisting capacity, *Dask* has made it possible to scale single-cell analysis pipelines to upwards of millions of cells [61]. In large-scale distributed environments, *i.e.* supercomputers, *Dask* has been deployed as a workflow manager for GPU-accelerated computation to predict protein structures from genomic sequences [62].

### The *Dask* framework

*Dask* [22] is a native Python framework that extends the SPE’s capabilities for multi-core and out-of-core computation. This allows *Dask* to scale beyond a single machine and fit increasingly large datasets into memory. By cloning the APIs of commonly used libraries like *NumPy*, *pandas* or *scikit-learn*, *Dask* minimizes the changes required to port pre-existing code [36]. The simplicity of *Dask* greatly reduces the barrier to entry for analysts that are new to distributed and parallel computing. This is especially important in domains such as computational biology and bioinformatics where data analysis pipelines are often developed by scientists without a formal computer science background using the SPE in local workstations.

**Fig. 2:**
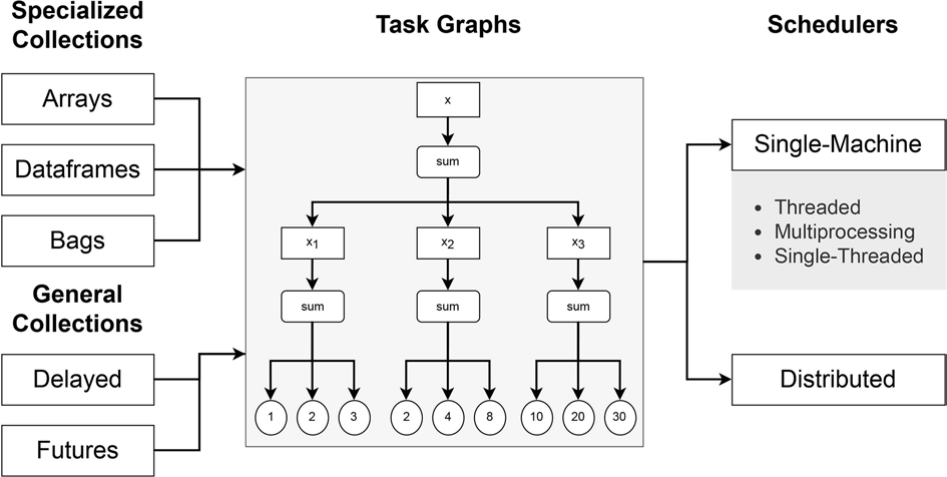
Main components of the *Dask* framework. Collections are processed by task graphs, which are executed by schedulers. In a task graph the nodes represent functions (f) and edges are Python objects (o)

The *Dask* framework combines blocked algorithms with task scheduling to achieve parallel and out-of-core computation. The framework is composed of collections, task graphs and schedulers. Collections are data structures that represent and organize the data. Task graphs and schedulers determine how computations are performed (Fig. 2).

*Dask* implements three collection types specialized in building task graphs for structured and unstructured data: Dask Arrays, Dask Dataframes and Dask Bags. Dask Dataframes are limited to two-dimensional tables, while Dask Arrays can have higher dimensionality. Dask Dataframes split the dataset into multiple partitions along the index. Each partition consists of a *pandas* Dataframe that can be processed independently, parallelizing the workload. Dask Arrays creates chunks by splitting *NumPy* arrays along the index and columns. Bags are a more flexible data type that can hold any combination of Python objects. They are particularly useful for working with unstructured data in tasks such as graph or text mining. Because it mimicks the interface of the *pandas* and *NumPy* libraries, *Dask* offers many of the same statistical and analytical functionalities.

In addition, *Dask* provides the more general Delayed and Futures interfaces that operate as building blocks for implementing custom data structures and algorithms in *Dask*. Like the aforementioned specialized collections, Dask Delayed executes task graphs lazily as needed, while Future executes functions eagerly and concurrently across multiple cores and machines.

To efficiently handle collections, *Dask* builds task graphs with blocked algorithms. Blocked algorithms are an approach to out-of-core, distributed computation. Blocked algorithms split data into smaller blocks that are loaded on demand and processed in parallel [63].

A task graph is a directed acyclic graph used to keep track of all tasks and their order. Each node is a function and the edges are objects generated by the preceding function node and used as input by the succeeding node (Fig. 2). Graph evaluation is performed lazily, meaning that task processing is delayed until computation is explicitly called. Due to the great flexibility of blocked algorithms, task graphs can interface with several problem assignment algorithms, such as task scheduling [64].

Task scheduling allows *Dask* to achieve parallelization by dividing the program into smaller tasks. For instance, when summing three partitions with three numbers each, the scheduler will assign one worker to each partition and process them in parallel. Once this done, the resulting smaller sums are aggregated into a final solution, Fig. 2.

In *Dask*, there are two groups of task schedulers: single-machine and distributed. Single-machine schedulers leverage local processes or threads, while distributed schedulers operate both locally and across multiple machines. While single-machine schedulers do not require setup and incur in a smaller overhead, distributed schedulers are more sophisticated and offer more features such as a diagnostics dashboard for monitoring performance.

Single-machine schedulers include the threaded scheduler, multiprocessing scheduler and single-threaded synchronous scheduler. The single-threaded scheduler has no parallelism, performing all computations in a single thread, which facilitates debugging. The threaded scheduler leverages a single machine’s entire thread pool, which allows for faster computation times for code that releases the GIL. Lastly, the multiprocessing scheduler provides parallelism by assigning tasks to local processes. This is useful for bypassing the GIL when it cannot be released, as is often the case with pure Python code. However, because inter-process communication is slower than threaded execution, the multiprocessing scheduler does not always reduce execution times.

While single-machine schedulers ship threads or processes to local thread or process pools directly, distributed schedulers launch and interface with computations through clients that assign tasks to *workers*. In *Dask*, one worker corresponds to one process, with access to a worker-specific subset of the thread pool. One of the workers is appointed as the master which will coordinate other workers and direct them to execute the individual tasks found in graphs. As tasks are completed, workers become free and are assigned to any remaining tasks until all tasks are executed.

The primary purpose of distributed schedulers is to parallelize computation in clusters. However, clients for interfacing with distributed schedulers can also be initialized locally. This might be desirable for a number of reasons. As previously mentioned, distributed schedulers grants users with access to a diagnostics dashboard which allows them to assess computational performance. Furthermore, distributed schedulers offer improved data locality. Data locality consists in the process of moving computations closer to where data is stored, which is often more efficient than the reverse operation. In such cases, the additional overheard caused by setting up a distributed scheduler locally may prove worthwhile. Lastly, the distributed scheduler can leverage additional worker resources, such as additional memory for the local processing of large datasets or extra CPUs for CPU-intensive computations.

## Methods

The source code for processing the data and performing all analyses can be found at: https://github.com/martaccmoreno/gexp-ml-dask.

### Assessing phenotype predictive model performance

Using an *XGBoost*-powered classifier [20] with default hyper-parameters, we compared the performance of the three strategies (Distributed Threads, Distributed Processes and SPE) in a single machine environment for several phenotype prediction tasks.

In all cases, expression matrices comprise *n* sample rows characterized by *f* gene feature columns; label vectors have length *n*. Because all preprocessing steps used *Dask*, we took advantage of the increase in parallelization whenever possible.

After transcriptome profiling, the BRCA gene expression datasets underwent FPKM normalization. The dataset has 1,205 samples with BRCA molecular subtype information available, profiled across 60,483 genes. Following feature selection of coding genes we obtained a second, smaller feature matrix, in which the number of genes was reduced to 19,564.

The LUAD/LUSC gene expression matrix contained 776 samples and 19,560 genes. These dimensions resulted from filtering for samples of individuals with information on the number of cigarettes smoked per day. Only coding gene features were selected. Gene expression quantification files for this dataset underwent FPKM and UpperQuartile normalizations.

Synthetic read counts for the SYNTH dataset were generated using *compcodeR* [65], an R package that generates simulated count data. Tweaks in the simulated data distribution were used to mimic different classes. Simulated counts and their respective classes were later subsampled and shuffled with a Python script. The resulting SYNTH dataset has 5,000 samples and 20,000 genes.

#### Generating datasets with different dimensionalities

For dataset subsampling two approaches were applied: (i) sample-wise subsampling, in which the top 20,000 genes with higher variance were selected as features, and a varying number of samples *n* (200, 600, and 1,205) was randomly sampled; (ii) feature-wise subsampling, in which the number of samples was fixed as 1,205 (the total), and varying the number of features *f* was select from among those with the highest variance (1,000, 20,000 and 40,000).

#### Cross-validation

As of the time of writing, *Dask* has no official CV support; the proposed solution is to parallelize computation using *joblib*. However, this only scales CPU usage and does not help with scaling memory. This means that while runtime will be reduced, memory usage will peak similarly as in SPE. We have developed our own custom CV script to use with Dask in tasks involving gene expression data, which can be found in the following repository: https://github.com/martaccmoreno/gexp-ml-dask. This implementation leverages Dask’s out-of-core capabilities for adding disk drive memory to total available memory.

### Hyper-parameter optimization

The performance of *Dask* and SPE in intensive optimization tasks was evaluated by performing an exhaustive grid search on the coding subset of the BRCA dataset (n1,205 $\times$ f19,564). To that end, a grid containing two values for each of three *XGBoost* hyper-parameters was transversed as many times as the total number of combinations (2 $\times$ 2 $\times$ 2 = 8). Training set performance was evaluated using 5 $\times$2 nested CV.

### Single-cell RNA-seq preprocessing

To assess how *Dask* performs and compares against SPE in handling scRNA-seq data, the following tasks were performed:

1. Log-normalization of the data (log-norm);
2. Identification and selection of highly variable gene features (feature selection);
3. Data scaling to shift the distribution to a mean of 0 and standard deviation of 1.

This pipeline was applied in a single machine environment using two frameworks, *Dask* and SPE, on single-cell transcriptomes sampled from esophagus tissue. The dataset comprised 87,947 samples and 24,245 genes. Smaller dataframes were derived by randomly choosing samples and features without replacement.

## Results

To highlight the relevance of the use of scalable data analysis frameworks, we benchmark and compare the performance of the *Dask* and SPE frameworks in two scenarios: (i) an end-to-end ML pipeline for phenotype prediction from cancer and synthetic bulk transcriptomic data, with and without hyper-parameter optimization; (ii) in the preprocessing single-cell RNA-seq data. All tests were performed on a laptop running Linux Ubuntu 20.04.2 LTS with a 500 GB SSD, 16 GB of RAM and a multi-core processor powered by four cores comprising two threads each, with core frequency of up to 4.6 GHz. or the *Dask* framework, we have opted to use the Distributed scheduler due to the advantages presented in the previous section. *Dask* Distributed is deployed using two configurations: (i) Distributed Threads (DT), which spawns a single worker with access to all eight CPU threads in the thread pool; and (ii) Distributed Processes (DP), which spawns four workers, each with access to two threads.

### Inferring phenotypes from cancer transcriptomic data

Cancer is a complex and heterogeneous disease driven by genetic alterations which often result in gene expression dysregulation [66, 67]. RNA profiling [26, 68] of tumoral tissues is widely used to uncover cancer gene signatures. Accurate diagnosis is essential to achieve the best clinical outcome [69]. Predictive models of cancer molecular subtypes have been developed from transcriptomic data [70–74], which can assist clinicians deliver specific tumor tailored treatments [75–79], as well as provide further cancer subtype stratification [80–82]. Prognostic signatures for assessing patient disease-free survival have been derived with ML from whole-transcriptome data in several tissues [81, 83, 84].

#### Supervised learning tasks

Transcriptome data from the Cancer Genome Atlas (TCGA) [85] was used to show how phenotypes can be predicted from tumor-specific gene expression levels. Classification and regression models were built with SPE and *Dask* (Distributed Threads and Distributed Processes) in various ways. The performance of the three approaches is compared in terms of minimum run time, peak memory usage and accuracy for different predictive tasks:

1. Classification of five breast cancer molecular subtypes from breast gene expression data sequenced by TCGA (BRCA);
2. Prediction of the number of cigarettes smoked per day in individuals diagnosed with lung cancer based on lung gene expression data from TCGA (LUAD/LUSC);
3. Binary classification of synthetic samples from read counts generated *in silico* using different distributions to mimic two distinct classes (SYNTH).

The minimum runtime and peak memory usage values for the three supervised learning tasks are shown in Table 2. Minimum runtime is the shortest execution runtime taken from three repetitions. This metric was chosen since fluctuations in runtime are usually caused by external factors, e.g. other processes running in the background, meaning that the smallest value approximates the true execution time for the code under evaluation. Memory usage reports the highest value recorded during execution.

For the full BRCA dataset, the DT configuration offered gains of at least 11.82% for peak memory usage and 39.4% in runtime compared to DP and SPE. Likewise, DT outperformed the other two methods for the coding subset of the BRCA datset with gains of at least 35.5% and 14.6% for peak memory usage and minimum runtime, as well as the LUAD/LUSC dataset with a minimum improvement in performance of 17.6% and 10.1% for those same metrics. The results for the SYNTH dataset presented a trade off: while DT offered gains of 9.0% for peak memory consumption, DP had a minimum runtime that was 36.2% lower.

#### Dataset dimensionality

**Fig. 3:**
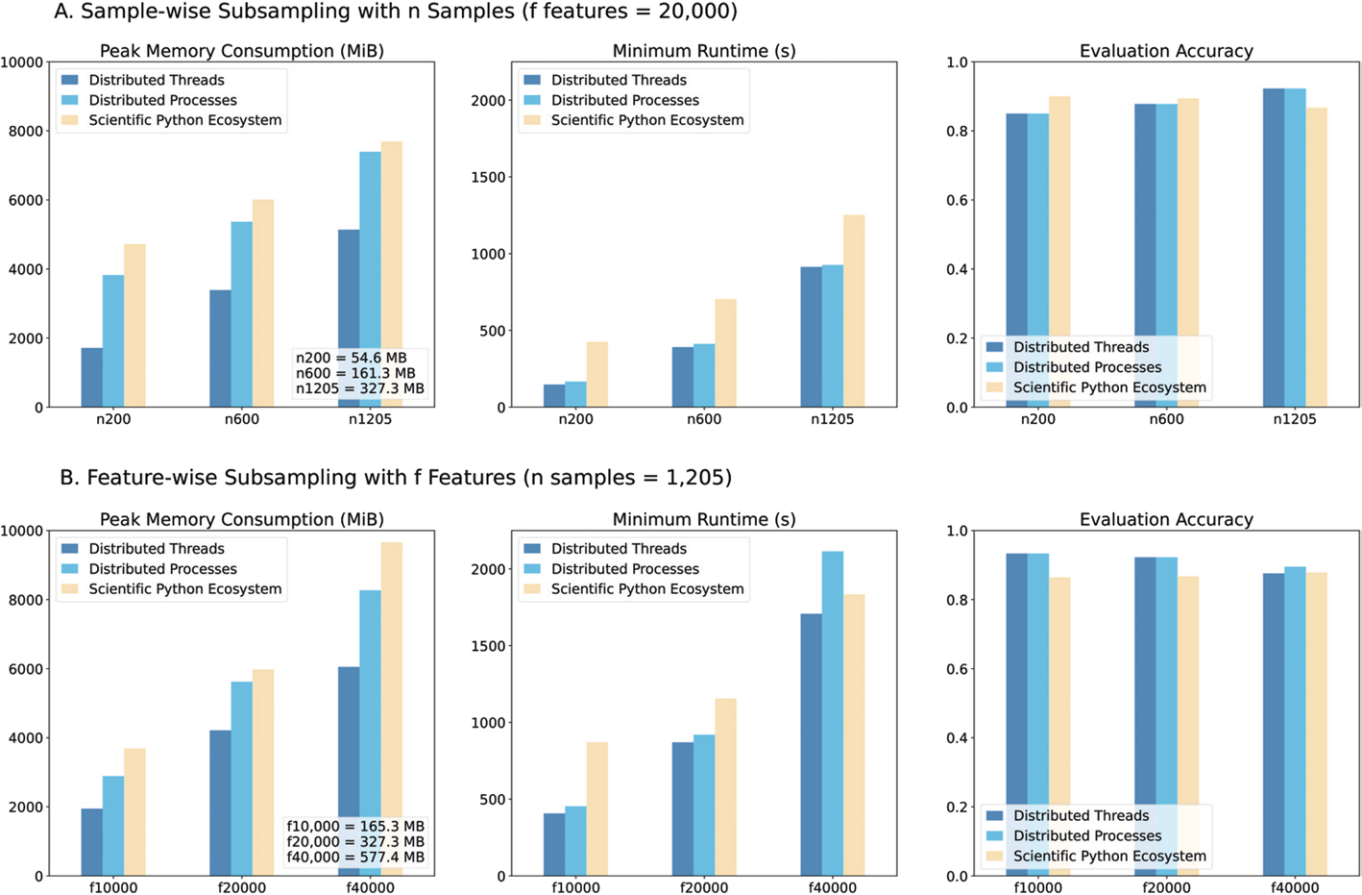
Performance comparison of the three strategies for the subsampled BRCA datasets. Peak memory, minimum runtime and classification accuracy were measured. Differences in accuracy resulted from the algorithmic implementation specificities of *Dask* and SPE. The data sizes shown in MB correspond to uncompressed tabular files

In addition to comparing performance for three datasets, we sought to test and compare the performance of the different strategies when applied to datasets with varying dimensionality, as they may be distinctively affected by a growing number of features (columns) or samples (rows). With subsampling we derived datasets of multiple sizes from the full BRCA dataset, see Fig. 3.

Overall, DT outperformed both DP and SPE in all cases, with the lowest peak memory usage and minimum runtime irrespective of dimensionality.

#### Intensive optimization tasks

Hyper-parameter optimization (HPO) performance was assessed for the three strategies using the coding subset of the BRCA dataset, see Table 2. DT outperformed the other methods for peak memory usage, with gains of at least 38.6%. Minimum runtime was similar for both *Dask* configurations, with gains of roughly 36% compared to SPE. All strategies exhibited similarly high levels of accuracy.

### Single-cell data analysis

Single-cell RNA sequencing (scRNA-seq) is enabling the study of gene expression at an unprecedented resolution to characterize complex tissues and disease [86]. The constant development of single-cell technologies resulted in an exponential growth in terms of cell profiled [87].

scRNA-seq computational analysis workflows follow a series of well-established steps [88]. Given the count matrices obtained by raw sequencing data alignment, the data is preprocessed before passing through downstream analysis [89]. Given the large amounts of data generated, scalable methods are essential for working with scRNA-seq data.

**Fig. 4:**
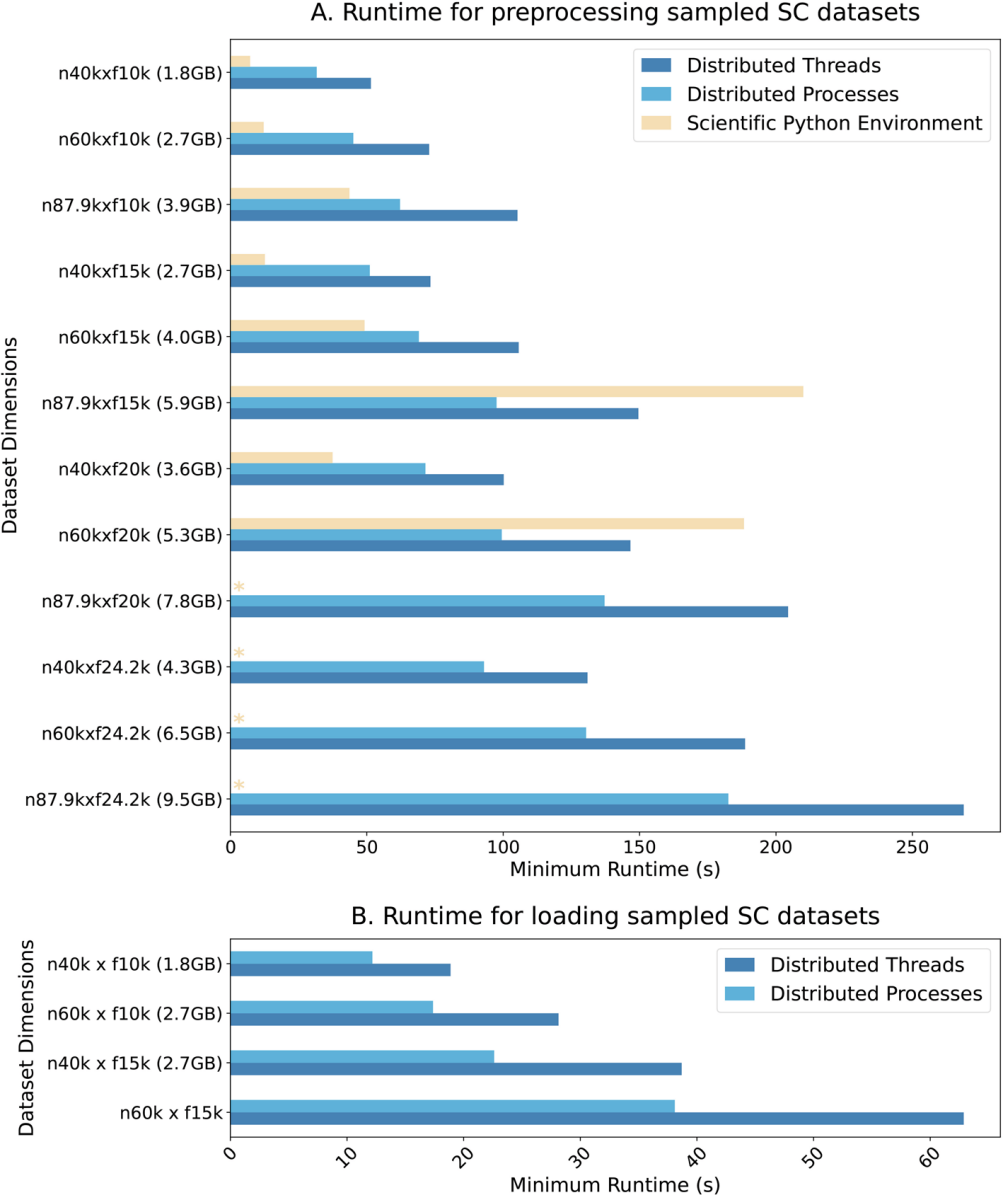
Runtime comparison between the *Dask* and SPE frameworks in **A** the preprocessing and **B** the full loading of scRNA-seq data. Datasets were subsampled for different dimensions. In **A**, both *Dask* Distributed configurations (Threads and Processes) partially load the data, processing dataset partitions; in **B**, the entire dataset is loaded. Asterisks represent instances when programs ran out of memory. n is the number of rows and f the number of features. The file sizes shown in GB correspond to uncompressed tabular files

Here, we show how *Dask* can scale several tasks (log-scale transformation, feature selection and scaling) commonly performed in the preprocessing of scRNA-seq data. Benchmarks are performed on a dataset sampled from esophagus tissue used to measure ischaemic sensitivity of human tissue at different time points [90]. The performance of DT and DP are compared with SPE relative to the minimum process runtime for different dimensionalities sampled from the complete dataset, see Fig. 4a.

In this scenario, for the lower dimensionality datasets, SPE outperforms both *Dask* Distributed configurations. However, for larger dimensiontionalities, both configurations surpass SPE and are, in fact, the only viable solution as SPE runs out of memory. Remarkably, the multiprocessing DP approach outperformed DT in all cases. To investigate this difference, we looked into the bottleneck for this simple pipeline: loading times (Fig. 4b).

Given that loading entire datasets into memory was not possible for higher dimensionalities, we tested DT and DP loading times on a subset of the available dimensionalities. As expected, loading comprises a considerable portion of runtime compared to the full pipeline (around 50%). DP always outperformed DT, meaning that even for the smallest dataset (n40k $\times$ f10k, with file size 1.8 GB), multiprocessing approaches greatly benefited loading times.

## Discussion

Getting started with *Dask* is simple, but knowing when and how to properly deploy it is vital for obtaining the best performance. As the results show, there are some important prerequisites to consider before choosing to scale computation with *Dask*.

Dataset dimensionality and learning complexity are the two major determinants of the analysis setup. Regarding the size of the dataset, one of the following scenarios can occur: (a) data fits comfortably in memory; (b) data cannot totally fit in memory unless disk drives are leveraged to expand total available memory; and (c) very large datasets that cannot be processed in a single machine. As for learning complexity, computational runtimes of data fitting are proportional to the complexity of the workflow. This step can be expedited with parallelization.

**Fig. 5:**
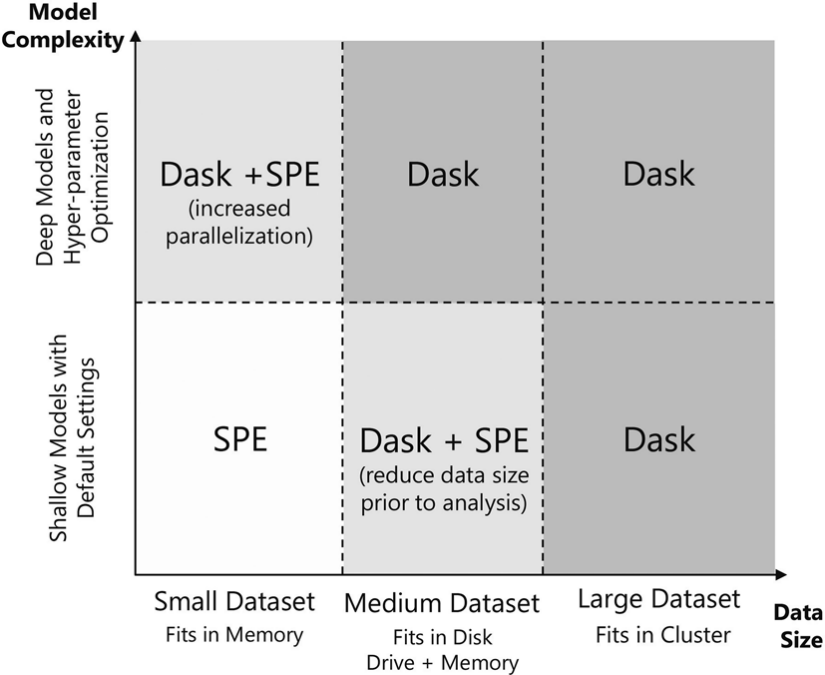
Proposed usage of SPE and *Dask* for different scenarios of model complexity and data size. When *Dask* and SPE are combined, *Dask* is used for preprocessing and SPE for data analysis and ML

Figure 5 shows different scenarios where SPE and *Dask* can be applied to surpass limitations and improve performance in ML models trained on datasets of different sizes, with or without intensive hyper-parameter optimization. Note that these guidelines are not strict. For example, if the size of large datasets can be reduced to fit in memory through pre-training processing with *Dask*, it may be more efficient to switch to SPE for the remainder of the computations.

### *Dask* usage guidelines for transcriptomics analysis

While integrating code with *Dask* requires minimal rewrites, to optimize performance it is important to consider the specificities of distributed computation explored in this paper, as well as the presented performance benchmarking results. In this section, we give usage guidelines based on our experience from working with transcriptomic data.

Transcriptome data is often presented as a singular tabular file. However, this format does not take full advantage of distributed computation. A naive approach would be to split data into several tabular files, enabling *Dask* to load and process each file as an independent partition to parallelize computation and reduce memory footprint. However, there are other file formats more suited for distributed computation, such as *Parquet* [91].

Parquet is a columnar file format that efficiently compresses stored data. It can easily store data as multiple files of fixed size that can be loaded as parallel partitions. Importantly, Parquet stores metadata information, which can greatly enhance *Dask*’s performance. This especially true of pipelines that include one of several operations that require knowing the index *a priori*, like sorting. Furthermore, metadata stores the data type of each column, which would otherwise have to be determined through a computationally expensive process of per-column sampling.

Partition size plays a significant role in performance. Ideally, partitions should be small enough to fit into each worker’s memory, but large enough to reduce the overhead caused by each additional partition. Ideal partition size depends on the characteristics and dimensions of each dataset. In our experiments, sizes between 60 to 100 MiB offered a good tradeoff. As several of the data analysis steps, like feature selection, reduce dataset size, distributing data across fewer blocks (repartition) and storing the result in memory (persist) can lead to more efficient task graph execution.

The choice between *Dask* Distributed Threads or Distributed Processes depends on the size of data, types of execution bottlenecks and proportion of GIL-bound code. Threads should be used to work with numeric collections (e.g. Arrays and Dataframes) that release the GIL. On the other hand, pure Python collections (e.g. Bags) are bound to the GIL and thus typically show gains in performance when leveraging several processes. For example, the data used for building predictive models in our supervised learning tests was small (under 1GB), but because the workflows mostly comprised highly vectorized computations, multithreading had a better overall performance. For the single-cell preprocessing pipeline, multiprocessing exhibited the best performance. This difference was also observed when testing loading times only. Since the file sizes for datasets varied between 1.8GB and 9.5GB, we posit that multiprocessing approaches generally outperform multithreading for larger data sizes and when loading times are an execution bottleneck.

## Conclusions

Genomic technology developments have led to an exponential increase in the volume of data collected with multiple molecular assays. The molecular characterization of cohorts of hundreds of individuals (e.g. GTEx [92], TCGA [85], Geuvadis [93]), of diverse cellular characteristics (e.g. ENCODE [94, 95] or the Roadmap Epigenomics Project [96]), and thousands of single cells [88, 97] has been achieved. This has prompted breakthrough advances in many bioinformatics topics, including precision medicine, cancer genetics, population genomics, and developmental molecular biology.

ML appears as a critical tool to map the relationship between the state of molecular entities and phenotypic traits. The magnitude and the high-dimensionality of transcriptomic data often requires considerable computational resources, in the realm of high-performance computing, which are not always available. Thus, alternative approaches for scalable machine learning and data analysis are required.

*Dask* is highly flexible and versatile and can be used as standalone tool or to support other frameworks. It facilitates scalable data analysis with multi-core and out-of-core computation functionalities. By cloning the API of commonly used scientific Python libraries, *Dask* makes its adoption rapid and seamless. Although we have focused on so-called shallow learning methods, *Dask* can also interface with Python Deep Learning libraries [60, 98].

Through several application examples we show that *Dask* can improve the performance of transcriptomics data analysis and scale computation beyond the usual limits. We foresee that frameworks like *Dask* will become an essential part of the computational data scientist’s toolkit, alleviating the burden of technical implementation and allowing researchers to concentrate on scientific questions.

**Table 1:** Examples of Python tools and frameworks for scalable data science

Name	Description	Website	References
bodo.ai	Native Python framework that improves performance with automated parallelization and compiler optimization	https://bodo.ai/	NA
Dask	Framework that parallelizes SPE data science operations with a familiar API	https://dask.org/	[22]
Fugue	Unified interface for distributed computing running Pandas code on Spark and Dask without any rewrites	https://github.com/fugue-project/	NA
Koalas	Project that simplifies the use of *Spark* distributed dataframes by adopting *pandas*’ DataFrame API	https://koalas.readthedocs.io/	NA
Modin	Library for interoperating with scalable ML frameworks	https://modin.readthedocs.io/	[99, 100]
RAPIDS	Framework for simplified GPU data science	https://rapids.ai/	[60]
Ray	Framework for scaling compute-intensive ML pipelines	https://www.ray.io/	[101]
Scalable Dataframe Compiler	A tool for compiling *pandas* operations on dataframes to facilitate parallelization	https://github.com/IntelPython/sdc	[102]
Vaex	Standalone tool for visualizing data and performing statistical calculations	https://vaex.io/	[103]

**Table 2:** Summary of memory and runtime performance for the three strategies on different datasets

	Peak memory (MiB)			Minimum runtime (s)		
Framework	DT	DP	SPE	DT	DP	SPE
BRCA n1,205 $\times$ f60,483 (713.3 MB)	**12**,**380**	14,039	*OOM*	**984**	1623	*OOM*
BRCA Coding n1,205 $\times$ f19,564 (311.3 MB)	**2897**	5870	4489	**903**	1057	1183
LUAD/LUSC n911 $\times$ f19,564 (194.9 MB)	**2988**	5204	3628	**410**	456	561
SYNTH n5,000 $\times$ f20,000 (300.9 MB)	**11**,**120**	12,217	16,443	585	**373**	1552
BRCA Coding HPO n1,205 $\times$ f19,564 (311.3 MB)	**4208**	6854	10,368	**1469**	1478	2324

Tests were performed with and without intensive hyper-parameter optimization (exhaustive grid search). Bolded values represent the best-performing result for each dataset and metric pair. The data sizes shown in MB correspond to the uncompressed tabular files. *OOM* out of memory

## Data Availability

All source code used to generate the results used in this paper, including steps on how to obtain the data from public repositories, can be found at: https://github.com/martaccmoreno/gexp-ml-dask.

## Abbreviations

CPU: Central processing unit
CV: Cross-validation
DP: Distributed processes
DT: Distributed threads
GIL: Global interpreter lock
HPC: High-performance computing
HPO: Hyper-parameter optimization
ML: Machine learning
SPE: Scientific Python environment
scRNA-seq: Single-cell RNA sequencing
SL: Supervised learning
TCGA: The Cancer Genome Atlas
